# Oral trehalose supplementation improves resistance artery endothelial function in healthy middle-aged and older adults

**DOI:** 10.18632/aging.100962

**Published:** 2016-05-19

**Authors:** Rachelle E. Kaplon, Sierra D. Hill, Nina Z. Bispham, Jessica R. Santos-Parker, Molly J. Nowlan, Laura L. Snyder, Michel Chonchol, Thomas J. LaRocca, Matthew B. McQueen, Douglas R. Seals

**Affiliations:** ^1^ Department of Integrative Physiology, University of Colorado Boulder, Boulder, CO 80309, USA; ^2^ Division of Renal Diseases & Hypertension, University of Colorado Denver, Aurora, CO 80045, USA

**Keywords:** aging, trehalose, endothelium-dependent dilation, large elastic artery stiffness, oxidative stress

## Abstract

We hypothesized that supplementation with trehalose, a disaccharide that reverses arterial aging in mice, would improve vascular function in middle-aged and older (MA/O) men and women. Thirty-two healthy adults aged 50-77 years consumed 100 g/day of trehalose (n=15) or maltose (n=17, isocaloric control) for 12 weeks (randomized, double-blind). In subjects with Δbody mass<2.3kg (5 lb.), resistance artery endothelial function, assessed by forearm blood flow to brachial artery infusion of acetylcholine (FBF_ACh_), increased ∼30% with trehalose (13.3±1.0 vs. 10.5±1.1 AUC, P=0.02), but not maltose (P=0.40). This improvement in FBF_ACh_ was abolished when endothelial nitric oxide (NO) production was inhibited. Endothelium-independent dilation, assessed by FBF to sodium nitroprusside (FBF_SNP_), also increased ∼30% with trehalose (155±13 vs. 116±12 AUC, P=0.03) but not maltose (P=0.92). Changes in FBF_ACh_ and FBF_SNP_ with trehalose were not significant when subjects with Δbody mass≥2.3kg were included. Trehalose supplementation had no effect on conduit artery endothelial function, large elastic artery stiffness or circulating markers of oxidative stress or inflammation (all P>0.1) independent of changes in body weight. Our findings demonstrate that oral trehalose improves resistance artery (microvascular) function, a major risk factor for cardiovascular diseases, in MA/O adults, possibly through increasing NO bioavailability and smooth muscle sensitivity to NO.

## INTRODUCTION

Arterial dysfunction develops with advancing age and increases the risk for cardiovascular diseases (CVD) [1, 2]. Two key features of arterial aging that increase the risk for CVD include endothelial dysfunction, as characterized by reduced nitric oxide (NO)-mediated endothelium-dependent dilation (EDD), and stiffening of the large elastic arteries [3-5]. Although the mechanisms underlying these functional changes are incompletely understood, oxidative stress and inflammation have been implicated as key mediators [6-8]. As such, therapies that inhibit oxidative and inflammatory signaling with age may have the potential to improve arterial function and reduce CVD risk in healthy middle-aged and older (MA/O) adults.

The autophagy promoting disaccharide, α,α-trehalose (referred to as trehalose), is cytoprotective in lower organisms [9-13] and reduces oxidative stress and inflammation in numerous in vitro and in vivo rodent models of age-related diseases [14-17]. Furthermore, in rodents trehalose preserves vascular function in pro-inflammatory age-associated disease states [15, 17]. As such, trehalose is emerging as a novel therapy to inhibit oxidative stress and inflammation and restore vascular function in diseases of aging.

A recent study by our laboratory shows that four weeks of trehalose in drinking water restores NO-mediated EDD and reverses large elastic artery stiffness in old mice to levels observed in young, while having no effect in young animals [18, 19]. These improvements were associated with reduced arterial superoxide production and normalization of arterial inflammatory proteins [18, 19]. Together, our findings provide strong evidence that trehalose may be an effective therapy to protect against age-associated arterial dysfunction through inhibiting vascular oxidative stress and inflammation. However, the efficacy of this therapy in humans is unknown.

The aim of the present study was to translate our preclinical findings to MA/O adults. We hypothesized that oral trehalose supplementation would improve resistance (microvascular) and/or conduit artery NO-mediated EDD, and reduce large elastic artery stiffness in MA/O adults free from CVD. We further hypothesized that these functional improvements would be associated with reduced oxidative stress and inflammation. To test this, we conducted a randomized, double-blind, parallel group study in which the diets of 32 healthy MA/O adults were supplemented with 100 g/day of trehalose or maltose for 12 weeks.

Importantly, this dose is roughly equivalent to the amount of trehalose that improved endothelial function in old mice on a g/kg body mass/day basis [19]. NO-mediated EDD, large elastic artery stiffness, and circulating markers of oxidative stress and inflammation were assessed at baseline and after four (EDD and circulating markers only) and 12 weeks of supplementation.

## RESULTS

### Subject enrollment

**Figure 1:**
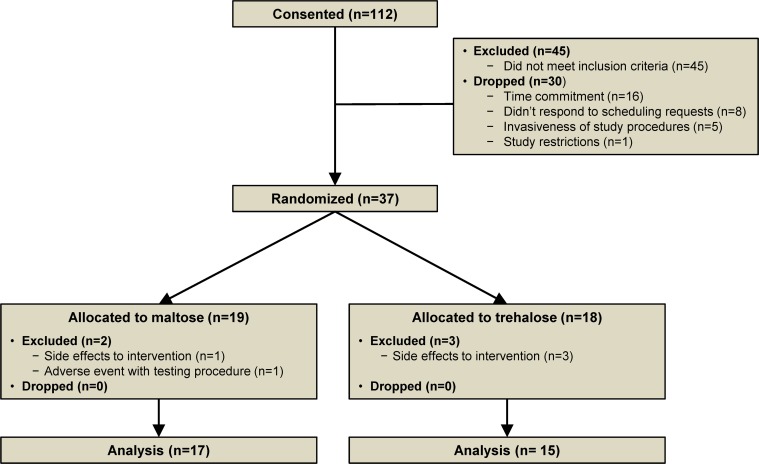
Study flow diagram

One hundred twelve subjects were consented for the study. Forty-five subjects did not meet inclusion criteria and 30 subjects opted out of the study prior to randomization due to the time commitment (n=16), study restrictions (n=1) and invasive testing procedures (n=5). An additional eight subjects never responded to scheduling requests. Nineteen subjects were assigned to the maltose group and 18 subjects to the trehalose group. Two subjects were excluded from the maltose group due to the development of side effects (n=1) and an adverse event related to a testing procedure (n=1). Three subjects were excluded from the trehalose group due to the development of side effects (Figure 1).

### Subject characteristics

There were no significant (P<0.05) group differences in age, gender, body mass, percent body fat, waist to hip ratio, systolic blood pressure, diastolic blood pressure, heart rate, maximal oxygen consumption, fasting glucose or total, high-density lipoprotein (HDL) or low-density lipoprotein (LDL) cholesterol between maltose and trehalose groups at baseline (all P>0.05, Table 1). There was a significant effect of time on body mass (F[2,27]=6.58, P=0.01) and this response did not differ between groups (P=0.87). In both the maltose and trehalose groups, there was a modest increase in body mass from baseline to week 4 (maltose: 72.8±3.4 vs. 72.1±3.4 kg, P=0.04; trehalose: 74.1±4.8 vs. 73.4±4.7 kg, P=0.02) but not from week 4 to week 12 (both P>0.05). Changes in body mass during the 12-week intervention ranged from −2.6 to 3.6 kg. Importantly, percent body fat did not change significantly across the intervention (F[1,29]=2.49, P=0.13). Moreover, no subject covariates changed differentially in the maltose and trehalose treated groups (all P>0.1, Table 1).

**Table 1:** Subject Characteristics

	Maltose			Trehalose		
Characteristic	Baseline	Week 4	Week 12	Baseline	Week 4	Week 12
N (men/women)	17 (8/9)	---	---	15 (7/8)	---	---
Age (years)	63±2	---	---	64±2	---	---
Mass (kg)	72.1±3.4	72.8±3.4*	73.0±3.3*	73.4±4.7	74.1±4.8*	74.4±4.8*
Fat (%)	30.3±1.9	---	31.5±2.0	31.2±1.9	---	31.4±1.9
Waist to hip ratio	0.83±0.02	---	0.84±0.02	0.84±0.02	---	0.84±0.03
Systolic BP (mmHg)	125±4	---	126±4	129±4	---	130±4
Diastolic BP (mmHg)	71±2	---	70±2	71±3	---	70±3
Pulse Pressure (mmHg)	51±4	---	54±4	53±3	---	56±3
Heart rate (beats/min)	56±1	57±1	58±2	57±2	56±2	58±2
VO2 max (ml/kg/min)	31.2±1.7	---	30.9±1.7	27.4±2.4	---	28.9±1.7
Total cholesterol (mg/dL)	179±10	176±10	177±10	180±8	180±8	177±8
HDL cholesterol (mg/dL)	59±3	58±2	56±2	58±6	58±6	55±5
LDL cholesterol (mg/dL)	102±8	101±7	102±7	105±6	104±6	103±7
Glucose (mg/dL)	85±2	90±4	92±6	85±1	84±2	83±1

Data are mean ± SE;

* P<0.05 vs. baseline of same group

BP blood pressure, VO2 max maximal oxygen consumption, HDL high-density lipoprotein, LDL low-density lipoprotein.

### Estimated dietary intake

Total energy intake and the relative intake of carbohydrates, fats, and protein did not significantly differ between the maltose and trehalose groups at baseline (all P>0.1). There was a trend towards a significant effect of time on energy intake in both groups (F[1,26]=3.83, P=0.06). Relative carbohydrate intake was higher at week 12 vs. baseline in the maltose group (57±2 vs. 49±1% total calories, P<0.001) and there was a trend for carbohydrate intake to be elevated after 12 weeks of trehalose (55±2 vs. 50±3% total calories, P=0.07). In contrast, neither relative protein nor fat intake changed significantly across the intervention period (P>0.1, Table 2).

**Table 2:** Dietary Intake

	Maltose		Trehalose	
Characteristic	Baseline	Week 12	Baseline	Week 12
Daily energy intake (Kcal)	1719±143	2050±141	1874±175	1977±166
Daily relative carbohydrate intake (% total Kcal)	49±1	57±2‡	50±3	55±2
Daily relative protein intake (% total Kcal)	16±1	14±1	15±1	15±1
Daily relative fat intake (% total Kcal)	35±2	29±1.5	35±2	30±2

Data are mean ± SE;

‡ P<0.001 vs. baseline of same group. Kcal kilocalories.

### Resistance artery EDD

Forearm blood flow (FBF) values were log transformed to conform to normality assumptions; area under the dose-response curve (AUC) was calculated from the transformed values. FBF to brachial artery infusion of acetylcholine (FBF_ACh_ AUC) did not differ between groups at baseline (P=0.90) and did not change differentially across the intervention period with maltose and trehalose supplementation (F[1,23]=2.03, P=0.17).

**Figure 2:**
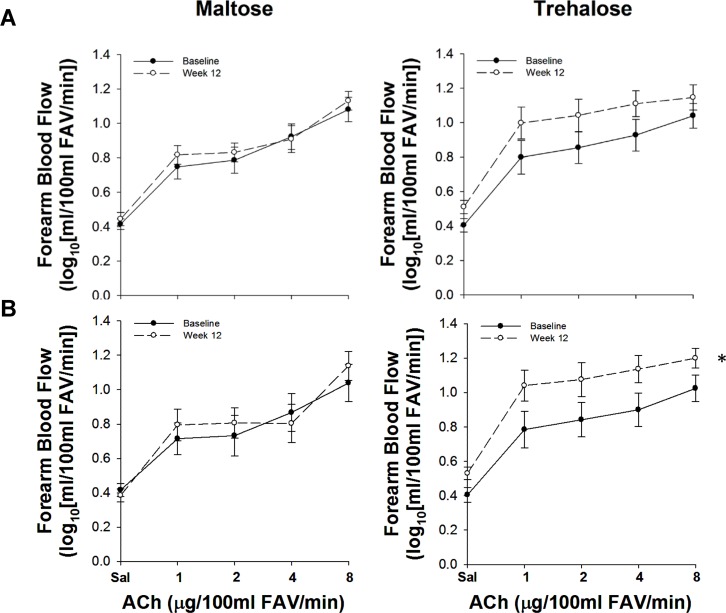
Forearm blood flow responses to acetylcholine (ACh) at baseline (closed circles) and following 12 weeks (open circles) of maltose and trehalose supplementation in all subjects (**A**) and in the subset of subjects who maintained body mass within 2.3 kg (**B**). FAV, forearm volume. Values are mean ± SE; *P<0.05 vs. baseline.

Because changes in body mass independently influence EDD [20], and weight gain was observed during the intervention period in both groups, we assessed the relation between the change in body mass and the change in FBF_ACh_ across the intervention period. The change in body mass from baseline to week 12 was inversely associated with the change in FBF_ACh_ (r=−0.41, P=0.04). To determine if this association was masking a treatment effect, we used multiple linear regression to assess the relation between group randomization and the change in FBF_ACh_ while controlling for the change in body mass. In this model, there was a trend for group randomization to be an independent predictor of the change in FBF_ACh_ from baseline to week 12 (P=0.09), suggesting a possible treatment effect independent of body mass. To isolate further the effect of trehalose treatment on resistance artery EDD, we conducted a per-protocol analysis that included subjects who maintained their body mass within 2.3 kg (5 lbs.; Table 3); there were no baseline differences between these subgroups and no subject covariates changed differentially between subgroups with the intervention. Contrastingly, FBF_ACh_ did change differentially with maltose and trehalose treatment (F[1,19]=5.01, P=0.04) in this cohort. FBF_ACh_ was ∼30% greater after 12 weeks of trehalose (13.3±1.0 vs. 10.5±1.1 AUC, P=0.02), whereas there was no change with maltose supplementation (P=0.40, Figure 2).

**Table 3:** Subject Characteristics in the Subset of Subjects who Maintained Body Mass within 2.3 kg

	Maltose			Trehalose		
Characteristic	Baseline	Week 4	Week 12	Baseline	Week 4	Week 12
N (men/women)	14 (7/7)	---	---	12 (6/6)	---	---
Age (years)	64±3	---	---	65±2	---	---
Mass (kg)	71.3±4.0	71.6±4.0	71.6±3.9	72.7±5.1	73.3±5.1*	73.6±5.1
Fat (%)	29.1±1.8	---	30.0±1.8	30.6±2.4	---	30.9±2.4
Waist to hip ratio	0.84±0.02	---	0.84±0.02	0.86±0.03	---	0.86±0.03
Systolic BP (mmHg)	125±4	---	127±4	131±4	---	131±4
Diastolic BP (mmHg)	71±2	---	70±2	72±3	---	70±4
Pulse Pressure (mmHg)	51±5	---	57±4*	55±4	---	56±4
Heart rate (beats/min)	57±1	57±1	56±2	56±2	56±2	59±2
VO2 max (ml/kg/min)	31.8±1.8	---	31.5±1.8	29.7±2.1	---	29.5±2.1
Total cholesterol (mg/dL)	180±12	177±12	175±12	183±9	187±9	183±10
HDL cholesterol (mg/dL)	60±3	59±3	57±3	56±6	57±6	53±5
LDL cholesterol (mg/dL)	103±10	101±9	99±9	109±6	111±5	111±7
Glucose (mg/dL)	85±2	88±5	91±7	84±1	83±2	84±2

Data are mean ± SE;

* P<0.05 vs. baseline of same group.

BP blood pressure, VO2 max maximal oxygen consumption, HDL high-density lipoprotein, LDL low-density lipoprotein.

### Resistance artery NO bioavailability

**Figure 3:**
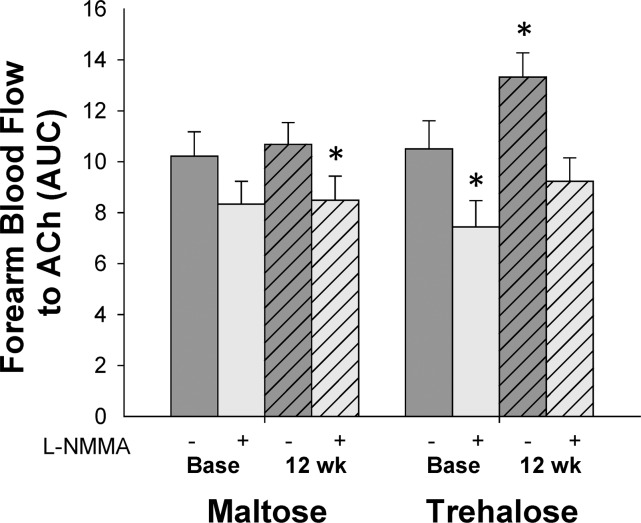
Forearm blood flow responses to acetylcholine (ACh) in the absence (dark grey bars) vs. presence (light grey bars) of the endothelial NO synthase inhibitor, NG-monomethyl-l-arginine (L-NMMA) at baseline (base) and following 12 weeks of maltose and trehalose supplementation in the subset of subjects who maintai-ned body mass within 2.3 kg. AUC, area under the dose response curve. Values are mean ± SE; *P<0.05 vs. baseline FBF_ACh_ in the absence of L-NMMA.

In subjects with a change in body mass <2.3 kg, the increase in FBF_ACh_ following trehalose supplementation (expressed as AUC, Figure 3) was abolished when endothelial NO production was inhibited by co-infusion of L-NMMA (P=0.33 vs. baseline FBF_ACh_ in the absence of L-NMMA), demonstrating that improvements in FBF_ACh_ were mediated by increased NO bioavailability.

### Oxidative stress-mediated suppression of resistance artery EDD

**Figure 4:**
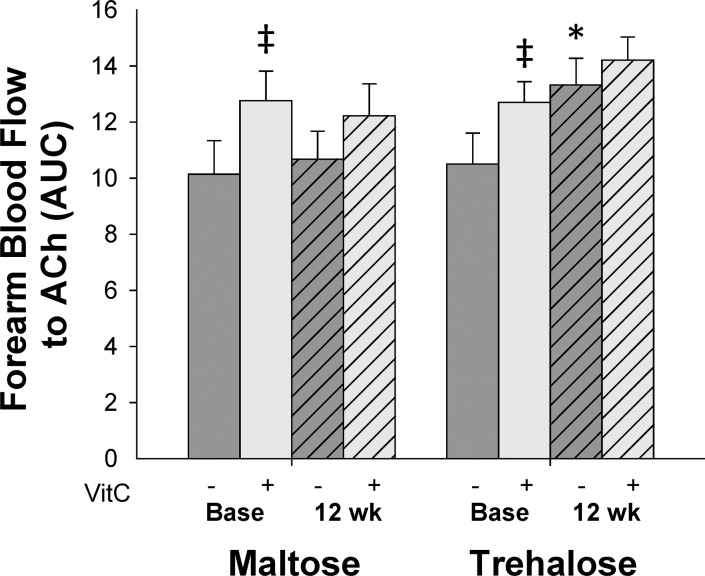
Forearm blood flow responses to acetylcholine (ACh) in the absence (dark grey bars) vs. presence (light grey bars) of the antioxidant, vitamin C, at baseline (base) and following 12 weeks of trehalose and maltose supplementation in the subset of subjects who maintained body mass within 2.3 kg. AUC, area under the dose response curve. Values are mean ± SE; *P<0.05 vs. baseline of same group; ‡P<0.05 vs. forearm blood flow to ACh in the absence of vitamin C at the same time point.

In subjects in whom body mass changed <2.3 kg, the response of FBF_ACh_ to co-infusion of the antioxidant vitamin C did not change differentially across the intervention period (F[1,16]=0.06, P=0.80). Co-infusion of vitamin C improved FBF_ACh_ in both groups at baseline (maltose base: 12.8±1.1 vs. 10.1±1.2 AUC, P=0.02; trehalose base: 12.7±0.7 vs. 10.5±1.1 AUC, P=0.01), whereas no significant improvement was observed following the intervention period in either group (maltose post: 12.2±1.1 vs. 10.7±1.0 AUC; trehalose post: 14.2±0.8 vs. 13.3±1.0 AUC, both P=0.1, Figure 4).

### Resistance artery endothelium-independent dilation

FBF to sodium nitroprusside (FBF_SNP_), a measure of smooth muscle sensitivity to NO, did not differ between groups at baseline (P=0.12). In all subjects, there was a trend for FBF_SNP_ to change differentially following 12 weeks of maltose and trehalose treatment (F[1,23]=3.18, P=0.09).

**Figure 5:**
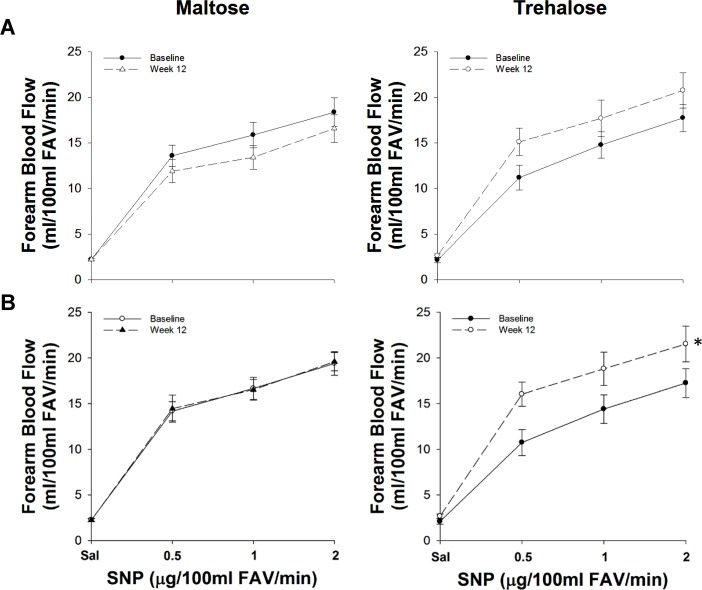
Forearm blood flow responses to sodium nitroprusside (SNP) at baseline (closed circles) and following 12 weeks (open circles) of maltose and trehalose supplementation in all subjects (A) and in the subset of subjects who maintained body weight within 2.3 kg (B). FAV, forearm volume. Values are mean ± SE; *P<0.05 vs. baseline.

The change in body mass from baseline to week 12 was inversely related to the change in FBF_SNP_ (r=−0.40, P=0.046). Moreover, group randomization was an independent predictor of the change in FBF_SNP_ when controlling for the change in body mass from baseline to week 12 using multiple linear regression (r=0.39, P=0.04), indicating an independent effect of trehalose treatment on smooth muscle sensitivity to nitric oxide. In the subset of subjects who maintained body mass within 2.3 kg, FBF_SNP_ changed differentially across the intervention period in the maltose and trehalose treated groups (F[1,19]=5.87, P=0.03). FBF_SNP_ increased ∼30% after 12 weeks of trehalose (155±13 vs. 116±12 AUC, P=0.03) whereas there was no change following 12 weeks of maltose supplementation (P=0.92, Figure 5).

### Conduit artery EDD

**Figure 6:**
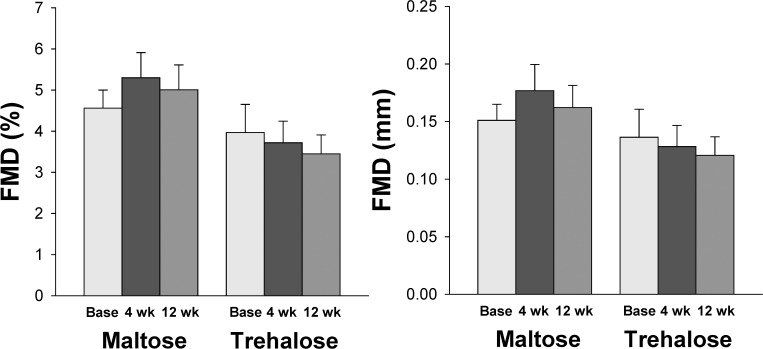
Brachial artery flow-mediated dilation (FMD) expressed as percent (left) and absolute (right) change at baseline (base) and following 4 and 12 weeks of maltose and trehalose supplementation. Values are mean ± SE.

Brachial artery flow-mediated dilation (FMD) was similar between groups at baseline (FMD (mm): P=0.60; FMD (%Δ): P=0.41). FMD did not change differentially across the intervention in the trehalose and maltose treated groups when FMD was expressed as either percent or absolute change (both P>0.1, Figure 6). In contrast to resistance artery EDD, the change in FMD after 4 and 12 weeks of supplementation was not related to changes in body mass at these time points (all P>0.1). Moreover, group randomization was not an independent predictor of the change in FMD from baseline to week 4 or baseline to week 12 when holding body mass constant using multiple linear regression (all P>0.1).

### Aortic stiffness

**Figure 7:**
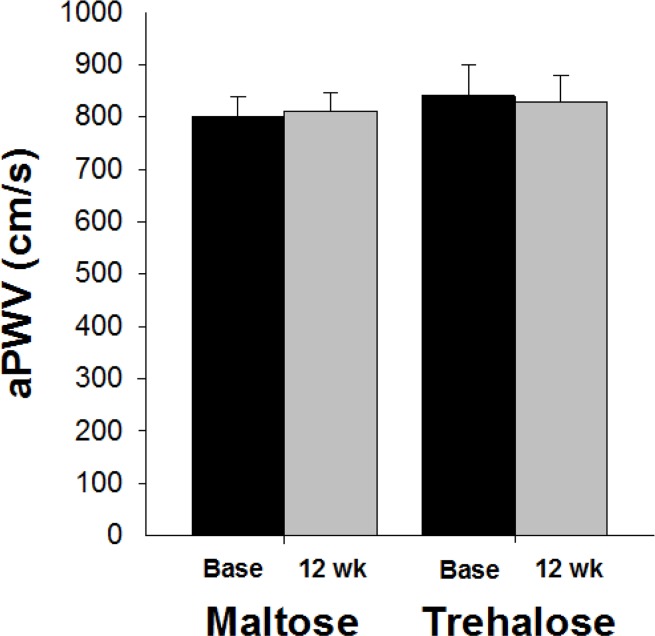
Aortic pulse wave velocity (aPWV) at baseline (base) and following 12 weeks of maltose and trehalose supplementation. Values are mean ± SE.

Aortic large elastic artery stiffness as assessed by aortic pulse wave velocity (aPWV) was not different between maltose and trehalose groups at baseline (P=0.56). There was no effect of time on aPWV (P=1.0) and aPWV did not change differentially across the intervention period in the trehalose and maltose groups (F[1,27]=0.31, P=0.59, Figure 7).

The change in body mass across the intervention was positively related to the change in aPWV (r=0.39, P=0.04). When holding body mass constant using multiple linear regression, group randomization was not an independent predictor of the change in aPWV (r=−0.11, P=0.56). In addition, aPWV did not change differentially across the intervention period in the cohort who maintained body mass within 2.3 kg (P=0.79).

### Carotid artery stiffness

**Figure 8:**
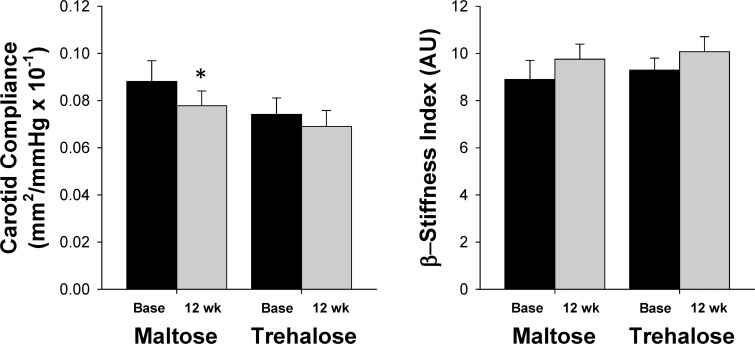
Carotid compliance (left) and β-stiffness index (right) at baseline (base) and following 12 weeks of maltose and trehalose supplementation. Values are mean ± SE. *P<0.05 vs. baseline of same group.

There were no baseline group differences in either carotid compliance (CC) or β-stiffness index (both P>0.1). CC changed significantly across the intervention period (F[1,26]=9.21, P=0.01) and this response did not differ between groups (F[1,26]=0.78, P=0.39). However, the change in CC with time was driven primarily by a decline in CC with maltose supplementation (0.078±0.006 vs. 0.088±0.009 mm^2^/mmHg × 10^−1^, P=0.03) as there was not a significant within-group change in CC with trehalose (P=0.12, Figure 8). This decline in CC with maltose treatment was likely due to the trend for increased PP observed in this group (Table 1), as no differences in β-stiffness index, a less blood pressure dependent measure of large elastic artery stiffness, was observed with either trehalose or maltose supplementation (both P>0.1, Figure 8). There was no relation between the change in body mass from baseline to week 12 and the change in either CC or β-stiffness index.

### Circulating factors

C-reactive protein (CRP), interleukin 6 (IL-6) and tumor necrosis factor alpha (TNF-α) were log transformed to meet normality assumptions. There were no baseline group differences in oxidized LDL, CRP, IL-6 or TNF-α and these factors did not change differentially across the intervention period in the maltose and trehalose treated groups (all P>0.1, Table 4).

**Table 4:** Circulating Factors

	Maltose			Trehalose		
Characteristic	Baseline	Week 4	Week 12	Baseline	Week 4	Week 12
Oxidized LDL (U/L)	46±5	42±4	42±3	45±3	44±4	46±4
C-reactive protein (log_10_[mg/L])	−0.21±0.08	−0.15±0.08	−0.23±0.09	−0.27±0.10	−0.16±0.13	−0.17±0.11
Interleukin-6 (log_10_[pg/mL])	−0.09±0.06	−0.04±0.06	−0.05±0.03	−0.04±0.07	−0.05±0.08	−0.07±0.07
Tumor necrosis factor-α (log_10_[pg/mL])	0.09±0.03	0.13±0.03	0.12±0.04	0.03±0.05	0.08±0.04	0.09±0.05

Data are mean ± SE.

Because body mass can independently influence these circulating factors [20-24], we determined the relation between the change in body mass and the change in each circulating factor from baseline to week 4 and from baseline to week 12. The only significant relation observed was a positive correlation between the change in body mass and the change in oxidized LDL after 12 weeks of supplementation (r=0.39, P=0.04). To determine if the association between weight gain and increased oxidized LDL might be masking a possible treatment effect, we used multiple linear regression to determine if there was an independent relation between group randomization (coded, bivariate variable) and the change in oxidized LDL from baseline to week 12, while holding the change in body mass constant. In this model, group randomization was not an independent predictor of the change in oxidized LDL (r=0.18, P=0.33), suggesting that trehalose did not have an independent effect on this circulating marker of oxidative stress.

### Side effects

The side effects reported included minor to moderate gastrointestinal discomfort (transient bloating, flatulence, and loose stools; maltose: n=0; trehalose: n=4) and changes in perceived energy levels (maltose: n=1; trehalose n=2). These side effects were expected, as they are characteristic of disaccharide consumption in general [13].

## DISCUSSION

This is the first study to examine the potential therapeutic effects of trehalose on any type of physio-logical function with age in humans. The present findings provide novel evidence that 12 weeks of oral trehalose supplementation improves resistance artery EDD, a measure of microvascular function, through increasing NO bioavailability in MA/O adults. Improvements in resistance artery endothelium-independent dilation were also observed with trehalose treatment, indicating increased vascular smooth muscle sensitivity to NO. In contrast, trehalose supplemen-tation had no effect on conduit artery EDD or large elastic artery stiffness. Together, these findings demonstrate that oral trehalose supplementation has heterogeneous effects on different functional aspects of arterial aging, and may be a novel strategy to improve resistance artery EDD in healthy MA/O adults. Because microvascular function (resistance artery EDD) is an independent predictor of incident CVD, these findings have important clinical implications for the primary prevention of CVD with advancing age.

### Trehalose supplementation and endothelial function

Resistance and conduit artery EDD are impaired with advancing age and are independent predictors of incident CVD [25-27]. As such, resistance and conduit artery endothelial dysfunction represent an intermediate phenotype in CVD progression [1, 2]. In the present study, there was no change in resistance artery EDD, as assessed by FBF to intra-arterial ACh, within the overall study population. However, resistance artery EDD improved ∼30% following 12 weeks of oral trehalose supplementation in subjects who maintained their body mass within 2.3 kg, suggesting that this intervention is effective for subjects who are able to maintain their baseline body mass despite the caloric content associated with the intervention. The increase in resistance artery EDD with trehalose supplementation was abolished by inhibition of endothelial NO production, indicating that this improvement was NO mediated. In contrast, maltose had no effect on resistance artery EDD or NO bioavailability. These findings are consistent with recent reports that trehalose has vascular-protective effects in age-related disease states in rodents [15, 17]. Moreover, these findings extend recent preclinical observations from our laboratory that oral trehalose supplementation improves NO-mediated EDD in response to ACh in old mice [19], and suggests that trehalose may be a viable therapy for the improvement of resistance artery EDD in MA/O adults.

In contrast to findings with resistance artery EDD, no changes were observed in conduit artery EDD, as assessed by brachial artery FMD, with trehalose supplementation. This remained true even when controlling for the modest increase in body mass during the intervention period. We did not anticipate this result considering that we previously found improvements in EDD in the carotid artery of mice with trehalose treatment [19]. This difference could be due to the stimulus used to induce EDD (pharmacological vs. mechanical) [28] in the mouse and human investigations. Specifically, improved EDD stimulated by ACh was observed in both mice and humans in different arterial beds, whereas there were no improvements in EDD stimulated by increased shear stress in MA/O adults. As such, it is possible that improvements in EDD with trehalose may be specific to non-mechanical stimuli and this will need to be delineated in future investigations.

The different findings with regards to resistance and conduit artery EDD are not surprising considering the heterogeneity in the structure, flow dynamics and microenvironment between conduit and resistance arteries [29-31]. While both resistance and conduit artery EDD predict incident CVD events [25-27], these measures are not related in MA/O adults free from CVD [28, 32]. Additionally, with advancing age impairments in resistance artery EDD are observed over two decades before impairments in conduit artery EDD, demonstrating differential effects of aging on the function of these arteries [33, 34]. Consequently, the different response observed for resistance and conduit artery EDD in the present study is in line with previous findings demonstrating dissociation between these two measures, and does not mitigate the importance of our findings that oral trehalose improves resistance artery EDD in MA/O adults. Resistance artery EDD alone is an important gauge of CVD health, as this measure independently predicts incident CVD and improves risk stratification for first major CVD event when added to the Framingham risk score [25]. Indeed, microvascular dysfunction is currently viewed as an early, critical antecedent to atherosclerotic CVD, preceding the development of macrovascular (conduit artery) dysfunction [35-37].

### Oral trehalose supplementation and endothelium-independent dilation

Endothelium-independent dilation is an important bioassay of smooth muscle sensitivity to NO. In the present study, trehalose improved resistance artery endothelium-independent dilation by ∼30% in subjects who maintained body mass within 2.3 kg. This increase in smooth muscle sensitivity to NO may have been an indirect effect of improved NO bioavailability with trehalose treatment, as NO is an important regulator of endothelium-derived vasoconstrictor factors [38-40].

The finding that trehalose improves endothelium-independent dilation in humans differs from previous findings in mice [19]. This may be due to fundamental differences between these experimental models. Specifically, endothelium-independent dilation is not impaired with aging in C57BL/6 mice [41-43]. As such, it may not have been possible to detect improvements in this outcome. In contrast, findings in humans are less consistent with some studies reporting modest impairments in endothelium-independent dilation with aging, especially in individuals with CVD risk factors [44]. In agreement with these reports, there was a trend for endothelium-independent dilation to be lower in subjects randomized to trehalose treatment at baseline, and this may partially explain the conflicting observations in mice and humans.

### Trehalose treatment and large elastic artery stiffness

Large elastic arteries become stiffer with age in the absence of disease [4, 45]. The resulting physiological sequelae from this phenotypic change include augmented systolic blood pressure and pulse pressure, microvascular remodeling, decreased coronary perfusion, and end organ ischemic tissue damage [46, 47]. Consistent with these observations, large elastic artery stiffness is an independent predictor of incident CVD in healthy MA/O adults [48, 49].

The mechanisms by which large elastic arteries stiffen with age involve both structural and functional changes [2]. Structural changes are characterized by increased expression of the load-bearing protein collagen and its cross-linking by advanced glycation end products, as well as the fragmentation and degradation of the elasticity-conferring protein, elastin [7, 50, 51]. Functional changes include increased smooth muscle tone, which, in turn, is influenced by endothelial release of vasodilator and vasoconstrictor factors [7, 51, 52]. We previously found that four weeks of oral trehalose supplementation reduced aPWV in old mice to levels observed in young. This was associated with reductions in collagen expression, suggesting that oral trehalose treatment promotes beneficial structural changes within the arterial wall in mice [18]. In addition, trehalose treatment restored endothelial function in old mice by increasing NO bioavailability [19], a key regulator of the functional component of large elastic artery stiffness with aging [53, 54]. In the present investigation, we found that 12 weeks of trehalose supplementation improved NO-mediated resistance artery EDD in MA/O adults, suggesting that trehalose also increases NO bioavailability in humans.

Despite promising preclinical evidence and the observed improvement in NO-mediated resistance artery EDD in the present study, trehalose had no effect on measures of aortic and carotid large elastic artery stiffness in healthy MA/O adults. These findings are in agreement with a growing body of literature demonstrating an inability of numerous nutrient-based interventions to reverse arterial stiffness in this population, despite promising results in preclinical models and/or in MA/O adults with cardiometabolic diseases. Such interventions include vitamin D [55], oral antioxidants [56-59], polyunsaturated fatty acids, folic acid, α-lipoic acid, soy, garlic [57] and a combination of resveratrol, tea extract, pomegranate extract, quercetin, acetyl‐l‐carnitine, lipoic acid, curcumin, sesamin, cinnamon bark extract, and fish oil [60]. While there are some conflicting reports supporting the efficacy of nutrient-based supplements for the reduction of large elastic artery stiffness in healthy MA/O adults, these investigations have either **a)** only assessed indirect measures of arterial stiffness that are more dependent on smooth muscle cell tone than arterial remodeling [61-63] or **b)** incorporated interventions lasting multiple years [64]. One exception to this is an investigation showing improved aPWV with isoflavone supplementation [65]. As such, the majority of current literature supports the conclusion that short term, nutrient based interventions have, at best, a minimal therapeutic effect on age-associated large elastic artery stiffness in humans.

### The effect of trehalose supplementation on systemic oxidative stress and oxidative stress-linked suppression of endothelial function

Oxidative stress develops with advancing age and inhibits NO-mediated EDD through the direct scavenging of NO and by damaging the enzymes and cofactors required for NO synthesis [66-70]. Oxidative stress also induces structural changes in the arterial wall, including increased collagen deposition, decreased elastin and augmented advanced glycation end product formation [7, 71]. Accordingly, acute inhibition of oxidative signaling can restore EDD and large elastic artery stiffness in healthy MA/O adults [69, 72, 73].

In lower organisms and ex vivo models, trehalose seems to protect against reactive oxygen species [9, 15] and oral trehalose supplementation decreases markers of oxidative stress in a mouse model of Parkinson's disease [16]. In the preclinical study previously conducted by our laboratory, four weeks of trehalose supplementation reversed age-associated increases in aortic superoxide production and abolished the oxidative stress-mediated suppression of EDD observed in old control mice [19]. In the present investigation there was no change in circulating oxidized LDL, a marker of systemic oxidative stress, with either trehalose or maltose supplementation. However, co-infusion of the antioxidant, vitamin C, increased FBFACh at baseline but not after trehalose treatment. This observation suggests that a reduction in oxidative stress may be a mechanism underlying the improvement in resistance artery EDD observed with trehalose, in accordance with previous findings in mice.

### The effect of oral trehalose supplementation on systemic inflammation

Age-associated pro-inflammatory signaling stimulates endothelial activation, cellular oxidant production, accumulation of cell damage, and alters autocrine and paracrine signaling [19, 75, 76]. Together, these changes disrupt cellular homeostasis [44, 77] and promote arterial remodeling [78, 79], making inflammatory signaling a key mechanism of vascular dysfunction with age. Accordingly, short term inhibition of inflammatory signaling increases EDD and decreases aPWV in MA/O adults, while having no effect in young adults [77, 80].

Although we observed decreased aortic expression of pro-inflammatory markers in old mice following trehalose supplementation, no changes in the circulating pro-inflammatory markers CRP, IL-6 and TNF-α were observed with trehalose treatment in the present investigation. This incongruity may be due to assessing inflammation in the vasculature in mice versus the circulation in humans, as the expression of inflammatory markers in these two tissues do not always correspond during healthy aging [75, 81].

### Limitations

The primary limitations of the present study are related to the challenges associated with a nutritional intervention. We chose to administer trehalose at a body weight equivalent dose to that which improved EDD in old mice to optimize the chances of seeing a therapeutic effect of trehalose on arterial function. However, there is a relatively high caloric content associated with 100 g/day of trehalose (∼400 Kcals/day) and despite nutrition counseling every two weeks, not all subjects were able to maintain their body weight over the 12 week intervention period or keep the macronutrient composition of their diet constant. The dose of trehalose administered also resulted in some transient gastrointestinal discomfort, as has been reported previously with the consumption of disaccharides [13]. Together, these factors limit the feasibility of administering a higher oral dose of trehalose or extending the intervention period in older adults to promote a larger treatment effect. Accordingly, future investigations with alternative methods of trehalose administration that increase bioavailability and/or enable longer intervention periods are warranted.

### Conclusions

Twelve weeks of oral trehalose supplementation improves NO-mediated EDD and smooth muscle sensitivity to NO in resistance arteries of MA/O adults, while having no effect on conduit artery EDD or large elastic artery stiffness. These findings provide novel evidence that trehalose may be an effective intervention for reversing microvascular dysfunction and decreasing CVD risk in healthy MA/O adults. The caloric content associated with trehalose supplementation poses a challenge for the broad public health application of this intervention.

## METHODS

### Subjects

Thirty-two men and postmenopausal women aged 50-77 years were studied. All subjects were non-smoking adults free from clinical disease as assessed by medical history, physical examination, blood chemistries, electrocardiogram, resting blood pressure, and cardiovascular responses to a graded exercise test. Subjects refrained from all cardiovascular acting medications for 24 hours prior to testing and all other medications for 12 hours. All procedures were approved by the Institutional Review Board at the University of Colorado Boulder. The nature, risks and benefits of all study procedures were explained to volunteers and their written informed consent was obtained before participation in the study.

### Procedures

All testing was performed at the Clinical Translational Research Center (CTRC) at the University of Colorado Boulder following a 12-hour fast from food and caffeine and 24-hour abstention from exercise and alcohol. All women were post-menopausal as confirmed by absence of menstruation for >1 year and follicular stimulating hormone levels>40 IU/L [82].

### Trehalose administration

Subjects were randomized (1:1 ratio) to consume trehalose (100 g/day) or maltose (100 g/day) for 12 weeks in a double-blind fashion. Food grade trehalose and maltose were purchased from Hayashibara (Okayama, Japan). Maltose was chosen as a control condition because **1)** like trehalose, maltose is a disaccharide of glucose, **2)** maltose does not have the same antioxidant and anti-inflammatory properties and/or beneficial physiological effects as trehalose when administered at similar concentrations [11, 15, 83-85] and **3)** maltose provides an isocaloric control condition. To replicate the preclinical study conducted by our laboratory as best as possible, trehalose and maltose were administered orally dissolved in 12 ounces of water. Subjects were provided individual containers with either 100 g of trehalose or maltose for each day of the intervention period. Subjects were also given a graduated water bottle and were instructed to mix one container of sugar with 12 ounces of water each day. Subjects were allowed to consume the intervention drinks at their own pace over the course of the day. Adherence was documented by having subjects return empty and unused containers every two weeks during the 12-week intervention period. Every two weeks subjects also received in-person nutrition counseling by a dietitian at the Boulder CTRC to promote stability of diet and body weight throughout the intervention.

### Subject characteristics and circulating factors

Arterial blood pressure was measured in triplicate over the brachial artery during supine rest (Noninvasive Hemodynamics Workstation, Cardiovascular Engineering Inc.) at baseline and after the 12-week intervention. Waist and hip circumferences were measured by anthropometry, and percentage body fat was measured by dual-energy X-ray absorptiometry (DEXA, DXA-GE Lunar; software version 5.60.003) at these same time points. Aerobic fitness was assessed at baseline and after the intervention by indirect calorimetry during incremental treadmill exercise (Balke protocol) [77, 86]. Total, LDL and HDL cholesterol, and fasting glucose were measured using standard assays at the University of Colorado CTRC Core Laboratory at baseline and after 4 and 12 weeks of trehalose and maltose supplementation. At these same time points, plasma oxidized LDL, IL-6 and TNF-α were assessed by ELISA (oxidized LDL: ALPCO; IL-6 and TNF-α: R&D systems) and CRP was measured by immunoturbidimetry, as described previously [70, 77]. All blood samples were drawn from an intravenous catheter placed in the left antecubital fossa.

### Dietary analysis

Stability of dietary intake was estimated using 3-day diet records at baseline and during the last week of the intervention period [20]. Diet records were assessed using Nutrition Data System for Research. Prior to starting the intervention, subjects met with a bionutritionist at the Boulder CTRC. All subjects were instructed to maintain their current caloric intake and were given suggestions on how to reduce carbohydrate intake by roughly 400 Kcals, (the caloric content of the study drinks) based on their baseline diet record. The caloric content of the study drinks was included in the diet records recorded during the last week of the intervention period.

### Resistance artery EDD and endothelium-independent dilation

Resistance artery EDD and endothelium-independent dilation were assessed at baseline and after the 12 week intervention period using strain gauge venous occlusion plethysmography (AI6 Arterial Inflow System, D.E. Hokanson Inc., Bellevue, Washington) as described previously [41, 72, 86, 87]. Briefly, a mercury-silastic strain gauge was placed around the forearm and an inflatable cuff was placed at the wrist and upper arm. The upper arm cuff cycled between 0 and 60 mmHg to occlude venous outflow during 7-second cycles. Wrist cuffs were inflated to 250 mmHg for the duration of all FBF measures to exclude hand circulation. FBF was calculated during the last 1.5 minutes of each infusion period. FBF values are expressed as AUC. In all subjects, the brachial artery of the non-dominant arm was catheterized for infusions. Forearm volume was measured by water displacement and drug infusion rates were normalized per 100 ml of forearm tissue.

Resistance artery EDD was determined by measuring FBF to increasing intra-arterial doses of acetylcholine (FBF_ACh_; 1, 2, 4, and 8 μg/100 mL forearm volume/min, 3.5-4 minutes per dose) and endothelium-independent dilation was assessed by measuring FBF to increasing doses of intra-arterial sodium nitroprusside (FBF_SNP_; 0.5, 1, and 2 μg/100 mL forearm volume/min 3.5-4 minutes per dose). To determine the contribution of NO to resistance artery EDD, FBF_ACh_ was measured in the absence and presence (co-infusion) of the endothelial NO synthase inhibitor NG-monomethyl-L- arginine (L-NMMA; 5 mg/min during a 10-minute loading dose followed by a 1 mg/min maintenance dose). Oxidative stress-mediated suppression of resistance artery EDD was assessed by measuring FBF_ACh_ in the absence and presence of the antioxidant vitamin C (25 mg/min during a 10-minute loading dose at an infusion rate of 2.5 ml/min followed by a 0.5 ml/min maintenance dose).

### Conduit artery EDD

Conduit artery EDD was measured using brachial artery FMD as described previously [68, 72, 77]. Briefly, the change in brachial artery diameter following five minutes of forearm blood flow occlusion was measured by duplex ultrasonography (Xario, Toshiba; multi-frequency linear-array transducer). FMD measurements were reported as absolute and percent change in accordance with recent guidelines [88, 89]. FMD was assessed during screening to ensure enrollment only of subjects with impaired endothelial function (defined as FMD<7% [the mean value we observe in healthy adults <30 years within our laboratory]). In addition, FMD was assessed at baseline and after 4 and 12 weeks of trehalose and maltose supplementation because this measurement is non-invasive and dynamic; changes in FMD have been observed with interventions less than one week in duration [70, 77].

### Aortic stiffness

Aortic stiffness was determined at baseline and after 12 weeks of trehalose supplementation by aPWV using transcutaneous applanation tonometry of the carotid and femoral arteries with simultaneous ECG recording (Noninvasive Hemodynamics Workstation, Cardiovascular Engineering Inc.) [4, 48]. Briefly, the time delay (transit time) between the foot of the carotid and femoral pressure waves was determined using the R-wave of the ECG recording as a timing reference. aPWV was calculated as the distance between measurement sites divided by transit time of the arterial pulse wave.

### Carotid artery stiffness

Local stiffness of the carotid artery was determined ∼2 cm proximal to the carotid bulb using two separate indices: CC and β-stiffness index. CC is a measure of arterial distention relative to pressure that provides an indirect assessment of vascular stiffness (higher compliance being associated with lower stiffness), whereas β-stiffness index is a less blood pressure dependent estimate of carotid large elastic artery stiffness corrected for distending pressure [90]. CC and β-stiffness index were determined by high-resolution ultrasonography (Xario, Toshiba; multi-frequency linear-array transducer) and subsequent applanation tonometry of the right common carotid artery (Noninvasive Hemodynamics Workstation, Cardiovascular Engineering Inc.) at baseline and following 12 weeks of trehalose and maltose supplementation. CC was calculated as described previously [91, 92] using the equation: cc = π × DD^2^ × (ΔD / DD) / (2 × PP) where DD is diastolic diameter, ΔD is the change in diameter across the cardiac cycle and PP is carotid artery pulse pressure. β-stiffness index was calculated as described previously [91, 93] using the equation: β = (Ln(SBP / DBP)) / (ΔD × DD) where Ln is the natural logarithm, SBP is systolic blood pressure and DBP is diastolic blood pressure.

### Data analysis

Statistical analyses were performed in SPSS (IBM SPSS Statistics 22). The Shapiro-Wilk test was used to assess normality and non-normal variables (CRP, IL-6, TNF-α, and FBF_ACH_) were log-transformed. Group differences at baseline were assessed using independent Student's t tests for between-group contrasts. Repeated measures ANOVA was used to determine group (maltose vs. trehalose) by time interactions for all clinical characteristics and primary outcome measures. In the case of a significant interaction or significant overall effect of time, a paired samples t-test for within-group contrast was performed with Bonferroni correction. The linear relation between variables of interest was assessed using Pearson product-moment correlation analyses. Multiple linear regression was used to evaluate the independent relation between group randomization (coded, bivariate variable) and the change in primary outcomes across the intervention period, while controlling for other factors. Statistical significance was set at P<0.05.

To gain insight into the independent effect of the intervention on changes in vascular function, endpoints were assessed in all subjects (in accordance with intention to treat principles), as well as a subset that adhered to the protocol and maintained body mass within 2.3 kg (5 lbs., i.e. ≤5% of total body mass for all patients included in the analysis). This cut point was chosen as changes in body mass of greater magnitude (without modifications in other lifestyle factors) are associated with alterations in endothelial function and large elastic artery stiffness [20, 94, 95].
